# Including emergency and acute care as a global health priority

**DOI:** 10.1186/1865-1380-4-75

**Published:** 2011-12-12

**Authors:** Nicholas Risko, Emilie JB Calvello, Sarah Stewart de Ramirez, Mayur Narayan, Jon Mark Hirshon

**Affiliations:** 1 University of Maryland School of Medicine, 655 W. Baltimore Street, Baltimore MD 21201, USA; 2 Department of Emergency Medicine, University of Maryland School of Medicine, Baltimore, Maryland 21201, USA; 3 Johns Hopkins School of Medicine, Department of Emergency Medicine, 600 N. Wolfe Street, Baltimore MD 21218, USA; 4 Department of Surgery, Trauma/Critical Care/Acute Care Surgery, R Adams Cowley Shock Trauma Center, University of Maryland School of Medicine, Baltimore, Maryland 21201, USA; 5 Department of Emergency Medicine, Department of Epidemiology and Public Health, University of Maryland School of Medicine, 10 South Paca Street, Rm 4S-127, Baltimore, Maryland 21201, USA

## Abstract

A recent important global meeting to set the international action agenda concerning non-communicable diseases (NCDs) failed to draw substantial attention from the emergency medical and surgical community. Advocacy efforts on the part of emergency clinicians should be increased to highlight the critical services we provide and create an approach to addressing NCDs with the most effective balance of preventive and acute care services. *Acute care*, which encompasses all frontline treatment services for sudden or unexpected injury or illness, can serve as a focal point for the development of the common language and body of research needed to draw the attention of global leaders and policy makers.

## Letter to the Editors

Recently, the United Nations (UN) General Assembly met in New York to discuss a topic of critical importance, the prevention and control of non-communicable diseases (NCDs). This meeting provided an important update to the 2000 World Health Assembly, which presented a global strategy to combat NCDs, resting upon the pillars of surveillance, primary prevention, and strengthened health care. Unfortunately, over the past decade limited advocacy from health-delivery fields has led to an agenda that emphasizes prevention while giving inadequate attention to strengthening health care. Though prevention is essential, acute care specialties that provide frontline treatment for sudden or unexpected illness or injury, like emergency medicine, need to take immediate and sustained action to highlight the importance of the services they provide. Aligning key players to support developing countries in planning for the best mix of acute and preventive services is an urgent priority with the potential to save and improve millions of lives.

We have many compelling reasons to get involved. The UN reports that 36 million people died from NCDs in 2008, representing 63% of the 57 million global deaths that occurred during that year. Eighty percent of the NCD deaths were caused by four conditions: cardiovascular diseases, diabetes, cancers, and chronic respiratory diseases. An increasing proportion of these deaths are occurring in developing countries as they move through the epidemiologic transition. UN projections show that by 2030, non-communicable diseases will cause five times as many deaths as communicable diseases worldwide [1,2].

Steps by the global community to combat NCDs have not adequately addressed the need to strengthen our ability to provide acute care. The WHO Framework Convention on Tobacco Control [3], the Global Strategy on Diet, Physical Activity and Health [4], the Global Strategy to Reduce the Harmful Use of Alcohol [5], and the 2008-2013 Action Plan for the Global Strategy for the Prevention and Control of Non-Communicable Diseases [6] are all evidence of this. The recent General Assembly presented a valuable opportunity for global decision makers to correct this oversight; however, it appears that acute care was once again crowded off the agenda.

The recently released UN Report of the Secretary-General on the prevention and control of NCDs [1] aggressively attacked acute care platforms that provide essential and life-saving care to millions. Remarkably, in sections devoted to strengthening health-system capacity and response, the report states: "Health-care services models should be transformed from acute emergency care to chronic lifelong care" (p 13). This presents a troubling lack of understanding on the part of global leaders about the importance of access to high-quality acute care. These two elements should not be viewed as mutually exclusive. In developing country settings, it is critical that we focus on the full spectrum of care, from prevention to acute treatment to chronic life care and rehabilitation, if we are to relieve suffering and save lives.

Our message should emphasize how acute care plays a crucial function in the simple prevention of death and disability that primary care is not positioned to provide. Additionally, it is critical that global leaders recognize that even with full preventive measures the need for access to high-quality acute care will remain. The four NCDs outlined as priorities by the WHO all contain presentations that require the life-saving tools and knowledge only available through emergency care services. In the United Kingdom, despite relatively easy access to primary and preventive care, statistics show a rate of 29 visits/100 people per year to Accident and Emergency Departments across the country, with a sizable number of these visits due to complications of NCDs [7]. Extrapolating this rate to developing countries would bring us to over a billion visits per year worldwide - *even in the presence of fully developed preventive and primary care services*. Furthermore, in many settings acute care facilities are the sole access point for both immediate injury care and for populations whose health is not adequately protected and monitored by a primary care safety net [8].

By developing core messages backed by research and experience, utilizing a common language, and increasing our interaction with the global health community, we can grow from clinicians into advocates, helping bring attention to the critical and permanent role acute care services play in health systems. The global dialogue on NCDs sparked by the recent UN assembly presents an opportunity for participation that we don't want to miss.

## Abbreviations

EMS: emergency medical services; NCDs: non-communicable diseases; WHO: World Health Organization.

## Competing interests

The authors declare that they have no competing interests.

## Authors' contributions

NR performed background research and had a primary role in drafting the manuscript. EJBC conceived of the manuscript and participated in its drafting and editing. SR provided guidance and participated in the drafting and editing of the manuscript. MN provided guidance and participated in the drafting and editing of the manuscript. JMH participated in the design and coordination of the research effort, and the drafting and editing of the manuscript. All authors read and approved the final manuscript.

## Authors' information

The *International Acute Care Research Collaborative *(IACRC), located within the University of Maryland Global Health Initiative, is dedicated to saving lives through improvement in the global access and quality of acute care services. This is accomplished through groundbreaking research and strategic advocacy efforts. All authors are members of the IACRC.
